# Electrocardiogram Heart Rate as a Predictor of Severity in Acute Alcohol-Related Pancreatitis With Alcohol Withdrawal Syndrome

**DOI:** 10.7759/cureus.11737

**Published:** 2020-11-28

**Authors:** Stalin Viswanathan, Dheeraj Jain, R Vinayagamoorthi, Murugesan S Gayathri

**Affiliations:** 1 General Medicine, Jawaharlal Institute of Postgraduate Medical Education and Research, Pondicherry, IND; 2 General Medicine, Indira Gandhi Medical College & Research Institute, Pondicherry, IND; 3 Biochemistry, Indira Gandhi Medical College & Research Institute, Pondicherry, IND; 4 Radiology, Indira Gandhi Medical College & Research Institute, Pondicherry, IND

**Keywords:** alcohol, acute pancreatitis, alcohol withdrawal syndrome, ecg changes, severity scoring

## Abstract

Background

The severity of acute alcohol-related pancreatitis (AAP) with alcohol withdrawal syndrome (AWS) has not been studied. Electrocardiogram (ECG) has not been used as a predictor of severity in patients with AWS and acute pancreatitis.

Objectives

The study aimed to determine whether the ECG heart rate (HR) could predict the severity of AAP; secondarily, whether AWS influenced the severity of AAP based on Acute Physiology and Chronic Health Evaluation (APACHE) II and Bedside Index for Severity in Acute Pancreatitis (BISAP).

Methods

Demographics, comorbid illnesses, AWS, biochemistry, ECG, arterial blood gases, and CT findings were noted in patients with AAP. The severity of pancreatitis was scored into mild, moderate, and severe based on CT. BISAP, APACHE II, and ECG heart rate-APACHE (E-APACHE) were compared in patients with and without AWS. A receiver operating characteristic curve was used to find the best predictor of severity.

Results

Among 138 patients (M=128), 94 had AWS. ECG changes (≥1) were seen in 50%. Patients with AWS were younger, had consumed alcohol for a shorter duration, had higher systemic inflammatory response syndrome (SIRS), APACHE II, and E-APACHE II scores. APACHE II and E-APACHE II correlated significantly with severity grading, HR, alcohol duration, and AWS. HR was the best predictor of severe pancreatitis; E-APACHE was the best predictor for moderately severe pancreatitis.

Conclusions

Mostly, AAP appears to be mild; >2/3^rds^ have AWS. ECG findings were seen in 50%. HR has not been previously studied in patients with both AAP and AWS and is an easy and inexpensive test to predict the severity of pancreatitis in this cohort.

## Introduction

Acute pancreatitis is an inflammatory disorder characterized by acinar cell destruction whose diagnosis is based on the presence of any two of the following: characteristic abdominal pain, elevated lipase or amylase (three times normal), and findings on abdominal imaging [1]. Alcohol withdrawal syndrome (AWS) is a neuropsychiatric syndrome that follows reduction or stopping alcohol [2]. Tachycardia, altered mental status, vomiting, and electrolyte disturbances that are seen in AWS may mimic the clinical features or complications of acute pancreatitis. Arrhythmias, conduction defects, ST-T changes mimicking myocardial infarction, and QT-interval changes have all been described in acute pancreatitis [3]. Only one study of severe acute pancreatitis was available wherein heart rate variability was described to predict complications such as infected necrosis [4]. It was surmised that an electrocardiogram (ECG) would give a more objective and accurate measure of the heart rate than that of the pulse rate measured by the physician at the time of admission. The heart rate is used in both the Acute Physiology and Chronic Health Evaluation (APACHE II) and Bedside Index for Severity in Acute Pancreatitis (BISAP) scores that are commonly used to score the severity in pancreatitis [1]. It was hypothesized that such scoring systems calculated based on the ECG heart rate would also differ from those calculated using the pulse rate, and thereby altering the severity scores in patients with acute pancreatitis. So, we embarked on this study to find out whether the ECG heart rate could predict the severity of acute alcohol-related pancreatitis (AAP) in patients with AWS.

## Materials and methods

Patients

This retrospective cross-sectional study was performed at Indira Gandhi Medical College and Research Institute, a government-funded teaching institution at Pondicherry, India. The study aimed to find out whether the ECG heart rate could predict the severity of AAP, and secondarily, whether alcohol withdrawal syndrome influenced the severity of AAP based on scoring systems such as APACHE II and BISAP. AAP was diagnosed and classified into mild, moderate, and severe based on the Revised Atlanta Classification 2012 [1]. Alcohol withdrawal syndrome was diagnosed by the presence of any two of the following: autonomic hyperactivity, tremors of the hands, nausea or vomiting, insomnia, transient hallucinations/illusions, anxiety, psychomotor agitation, and generalized tonic-clonic seizures [2]. Records of patients with a diagnosis of acute pancreatitis or acute on chronic pancreatitis admitted between 1 June 2016 and 31 May 2019 were searched for in the Medical Records Department. All patients with a history of alcohol consumption > 6 months and having any two of the following (new-onset acute abdominal pain suggestive of pancreatitis, elevated lipase >3 times and contrast-CT findings suggestive of acute pancreatitis) were taken as cases of alcohol-related acute pancreatitis. Patients without a history of alcohol consumption and the presence of cholelithiasis/choledocholithiasis on imaging were excluded from the study. Ethical approval for this study was obtained from the Institute Ethics Committee.

Data collection

ECGs were perused and the following parameters were noted: heart rate, PR-interval, QRS-interval and QTc-intervals, ST-segment changes (elevation or depression), T-wave changes and other abnormalities if any. Heart rate >100/min was considered as tachycardia, while ≤60/minute was taken as bradycardia. A short PR-interval was <120ms and prolonged PR-interval was >200ms [5]. QRS-interval> 110ms was considered as prolonged [6]. Bazett method was used to calculate QTc=QT/(√RR). A QTc >450ms in males and >460ms in females were considered as prolonged QTc [7]. Atrial and ventricular enlargement, poor r wave progression, and low voltage complexes were not considered as ECG changes related to acute pancreatitis. Demographics and history of chronic illnesses such as cirrhosis, chronic kidney disease, surgeries, blood transfusions, diabetes mellitus, and previous episodes of pancreatitis were documented. Alcohol intake, duration and type of beverage, concurrent alcoholic hepatitis and upper gastrointestinal bleed, smoking, past alcohol withdrawal, deaddiction, and Clinical Institute Withdrawal Assessment for Alcohol revised (CIWA-Ar) scores were noted. Pleural effusion on chest radiography, age, altered consciousness, blood urea nitrogen and systemic inflammatory response syndrome (≥2) were used to calculate the BISAP score [8]. BISAP and APACHE II scores were calculated using Qx Calculate v8.0.3.0 a mobile app. APACHE II scores were calculated using both the admission pulse rate and ECG heart rate (E-APACHE). The findings on abdominal imaging were used to calculate a CT severity index of pancreatitis and patients classified into mild, moderate, and severe pancreatitis based on scores of 0-3, 4-6, and 7-10, respectively. Clinical and laboratory findings were compared between the three groups. Patients were also grouped into those with and without AWS, to find out the association between AWS and AAP.

Statistics

IBM SPSS for Windows v22 (IBM Corp., Armonk, NY) was used to analyze data. Chi-square test (or Fisher’s exact test) was used to analyze categorical variables, while the t-test was used for continuous variables. Pearson’s correlation coefficient was calculated for severity grading, ECG heart rate, alcohol duration, and alcohol withdrawal syndrome with respect to APACHE II and BISAP scores. ROC curve was drawn with the three grades of pancreatitis being the state variable, and pulse rate, ECG heart rate, APACHE II and E-APACHE II taken as the test variables. The area under the curve (AUC) was calculated for each severity grade to find the best predictor. A P value of ≤0.05 was considered statistically significant.

## Results

There were 138 patients with males constituting 92.8% (n=128). Acute on chronic pancreatitis and alcoholic hepatitis was seen in 43 and 28 patients, respectively. Diabetes mellitus was seen in 31 and seven of these individuals presented with diabetic ketoacidosis. Cirrhosis and chronic kidney disease (CKD) were seen in eight and 12 patients, respectively. Forty-one were smokers. Ninety-four had alcohol withdrawal syndrome, with eight of them had complicated withdrawal (seizures and/or delirium tremens). The CT severity index was available only for 119 patients. ECG changes (≥1) were seen in 50%. Sinus tachycardia and sinus bradycardia were observed in 31 and 11 patients respectively. Patients with AWS were younger, had consumed alcohol for a shorter duration, had higher systemic inflammatory response syndrome (SIRS), APACHE II, and E-APACHE II score when compared to those without AWS (Table 1).

**Table 1:** Baseline characteristics, laboratory parameters, and complications in those with and without alcohol withdrawal syndrome AWS, alcohol withdrawal syndrome; APACHE, Acute Physiology and Chronic Health Evaluation; ECG, electrocardiogram; BISAP, Bedside Index for Severity in Acute Pancreatitis

	AWS (n=94)	No AWS (n=44)	P values
Gender, Males and females (n)	90 and 4	38 and 6	0.04
Age (years)	41.1±9.8	44.4±9.6	0.06
Age >60 (n)	4	1	0.56
Duration symptoms (d)	3.8±4.3	3.2±2.4	0.43
Duration of stay (n)	7.2±2.8	7.8±2.8	0.20
Ethanol (g)	90.5±44.9	84.8±59.4	0.53
Alcohol duration (years)	13.7±7.36	16.5±7.7	0.04
Smoking (years)	4.6±7.3	6.1±11.2	0.42
Diabetes mellitus (n)	15	16	0.007
Previous admissions (n)	46	15	0.10
Pulse rate (beats/min)	84.2±12.6	84.7±17.1	0.85
Mean arterial pressure (mmHg)	93±14.0	90.1±15.0	0.28
Respiratory rate (breaths/min	20.7±5.0	19.6±6.2	0.26
Palpable Liver (n)	21	3	0.02
Total bilirubin mg/dL	1.9±1.5	2.3±3.4	0.37
Aspartate aminotransferase IU/L	90±92.2	83.8±113.1	0.75
Alanine aminotransferase IU/L	68.0±114.9	47.8±49.7	0.32
Alkaline phosphatase IU/L	173.9±128.8	248.2±283.2	0.19
Protein g/dL	8.9±2.1	6.5±1.0	0.50
Prothrombin time prolongation (s)	12.1±28.9	16.4±7.8	0.06
Lipase (U/L)	403±216.9	405±289.1	0.96
Maddrey discriminant function	29.2±16.6	41.0±20.7	0.09
Hematocrit %	39.5±6.5	38.3±11.2	0.53
Total leukocyte count x10^9^/L	9.962.7±4.131.2	8.978±4.211.0	0.20
ECG heart rate /min	85.4±20.2	82.0±16.9	0.30
ECG tachycardia (n)	22	9	0.69
ECG bradycardia (n)	8	3	0.73
ECG PR interval	139.3±21.4	138.7±21.5	0.89
Short PR interval <120ms (n)	17	9	0.74
ECG QTc interval	427.9±27.7	427.6±23.4	0.94
Prolonged QTc interval (n)	17	8	0.98
Mild pancreatitis (n)	49	22	0.81
Moderate pancreatitis (n)	23	12	0.72
Severe pancreatitis (n)	9	4	0.92
Acute on chronic pancreatitis (n)	14	29	0.90
Systemic inflammatory response syndrome (n)	26	19	0.07
BISAP 0-2 (n)	92	43	0.95
APACHE II score	5.5±3.7	7.5±3.9	0.04
APACHE II ECG score	5.9±3.5	8.6±3.9	0.009
Infection (n)	3	3	0.33
Diabetic acidosis (n)	3	4	0.14
Upper gastrointestinal bleed (n)	4	3	0.53
Acute kidney injury (n)	10	3	0.43
Alcoholic hepatitis (n)	21	7	0.38
Intensive care unit stay (n)	9	4	0.92
Death (n)	0	4	0.03

The patient’s age and mean arterial pressure, APACHE and E-APACHE score, BUN, creatinine and acute kidney injury (AKI), and ECG PR-interval varied significantly among the various severity grades of pancreatitis (Table 2). 

**Table 2:** Clinical characteristics and laboratory investigations in patients with various grades of pancreatitis CIWA, Clinical Institute Withdrawal Assessment; ECG, electrocardiogram; BISAP, Bedside Index for Severity in Acute Pancreatitis; APACHE, Acute Physiology and Chronic Health Evaluation

N=119/138	Mild (n=71)	Moderate (n=35)	Severe (n=13)	Significance
Age (years)	40.8±10.1	43.3±9.4	49.2±8.7	0.01
Age>60 (n)	2	1	2	0.10
Duration of symptoms (d)	4.0±9.4	3.2±1.19	2.8±1.9	0.46
Duration of Stay (d)	7.4±2.8	8.1±2.9	7.2±2.5	0.47
Alcohol duration (years)	13.6±7.3	17.7±8.0	12.9±8.1	0.02
Ethanol (g)	87.1±54.2	88.3±49.1	84.341.2	0.97
Smoking (years)	4.8±7.5	4.3±9.2	6.5±13.1	0.16
Diabetes mellitus (n)	20	3	5	0.03
Cirrhosis (n)	2	4	2	0.24
Past history pancreatitis (n)	21	7	1	0.18
Temperature (F)	98.8±1.3	98.3±1.3	98.9±1.4	0.63
Pulse rate (beats/min)	83.2±15.8	85.5±10.8	87.4±16.4	0.49
Respiratory rate (breaths/ min)	20.1±6.3	20.0±4.7	21.2±5.4	0.76
Mean arterial pressure (mmHg)	89.9±14.2	92.5±9.0	86.3±12.9	0.09
Hematocrit(%)	39.7±8.6	36.6±7.9	37.8±7.5	0.18
Total leukocyte count (x10^9^/L)	9.457±3.871	9.722±4.368	11.361±6.163	0.34
Systemic inflammatory response syndrome (n)	22	10	7	0.22
BISAP Median score	4	8	6	
CT Severity index (Median)	1	2	4	<0.001
APACHE II score	4.3±2.9	7.5±3.8	9.5±3.5	<0.001
APACHE II ECG-Heart rate score	5.1±3.1	8.13±3.9	9.5±2.9	0.001
Blood urea nitrogen	4.3±1.6	6.46±5.3	7.67±4.6	0.01
Creatinine(mg/dL)	0.9±0.1	1.4±1.1	1.5±0.8	0.002
Acute kidney injury (n)	1	8	3	0.001
Upper gastrointestinal bleed (n)	5	2	0	0.60
Infection (n)	3	3	0	0.43
Diabetic ketoacidosis (n)	4	2	0	0.68
Bilirubin (mg/dL)	1.8±1.4	1.9±1.5	3.2±4.7	0.12
Aspartate aminotransferase (U/L)	83.4±105.9	78.3±81.1	114.8±120.2	0.54
Alanine aminotransferase (U/L	51.4±49.1	77.0±175.4	51.2±37.6	0.51
Prothrombin time prolongation(s)	14.1±8.6	9.9±8.4	11.8±5.3	0.20
Maddrey discriminant function score	35.0±20.9	24.7±13.5	47.2±19.2	0.08
Alcoholic hepatitis (n)	10	11	4	0.07
Severe alcoholic hepatitis (n)	5	4	2	0.52
Alcohol withdrawal syndrome (n)	49	23	9	0.93
CIWA score	17	9	3	0.89
Complicated withdrawal (n)	4	2	1	0.95
Chronic calcific pancreatitis (n)	3	0	0	0.35
Pancreatic Ascites(n)	0	5	6	0.001
Death (n)	0	1	3	<0.001
ECG heart rate	84.6±20.0	83.9±19.4	86.7±21.0	0.91
ECG tachycardia (n)	17	6	4	0.56
ECG PR	135.7±20.4	143.8±21.1	150.3±22.7	0.02
Short PR (n)	16	5	1	0.33
ECG QRS	92.0±10.5	90.7±9.9	89.7±8.0	0.67
ECG QTc	423.6±26.2	429.2±27.7	439.1±28.2	0.14
Prolonged QTc (n)	10	5	4	0.30

APACHE II and E-APACHE II score correlated significantly with the severity grading, ECG heart rate, alcohol duration, and alcohol withdrawal syndrome (Table 3).

**Table 3:** Correlation of severity grading, ECG heart rate, alcohol duration and alcohol withdrawal syndrome ECG, electrocardiogram; BISAP, Bedside Index for Severity in Acute Pancreatitis; APACHE, Acute Physiology and Chronic Health Evaluation

Variables	Correlating factors	Correlation; Significance (CI)
Severity grading	APACHE II	R=0.502; P <0.001(0.293-0.691)
	E-APACHE II	R=0.448; P <0.001 (0.180-0.663)
	BISAP score	R=0.348; P<0.001 (0.209-0.611)
	Alcoholic hepatitis	R=0.186; P <0.04 (0.004-0.239)
	Patient age	R=0.243; P<0.004 (0.100-0.404)
	Death	R=0.337; P<0.001 (0.191-0.543)
ECG heart rate	APACHE II	R=0.357; P=0.03 (0.058-0.632)
	BISAP	R=0.375; P=0.02 (0.018-0.703)
Alcohol duration (years)	ECG heart rate	R=0.332; P=0.04 (0.033-0.564)
	APACHE II	R=0.345; P=0.03 (0.053-0.593)
	E-APACHE II	R=0.357; P=0.03 (0.058-0.632)
Alcohol withdrawal syndrome	APACHE II	R=-0.243; P=0.04 (-0.523-0.44)
	E-APACHE II	R=-0.318; P=0.009 (-0.545-0.032)

**Figure 1:**
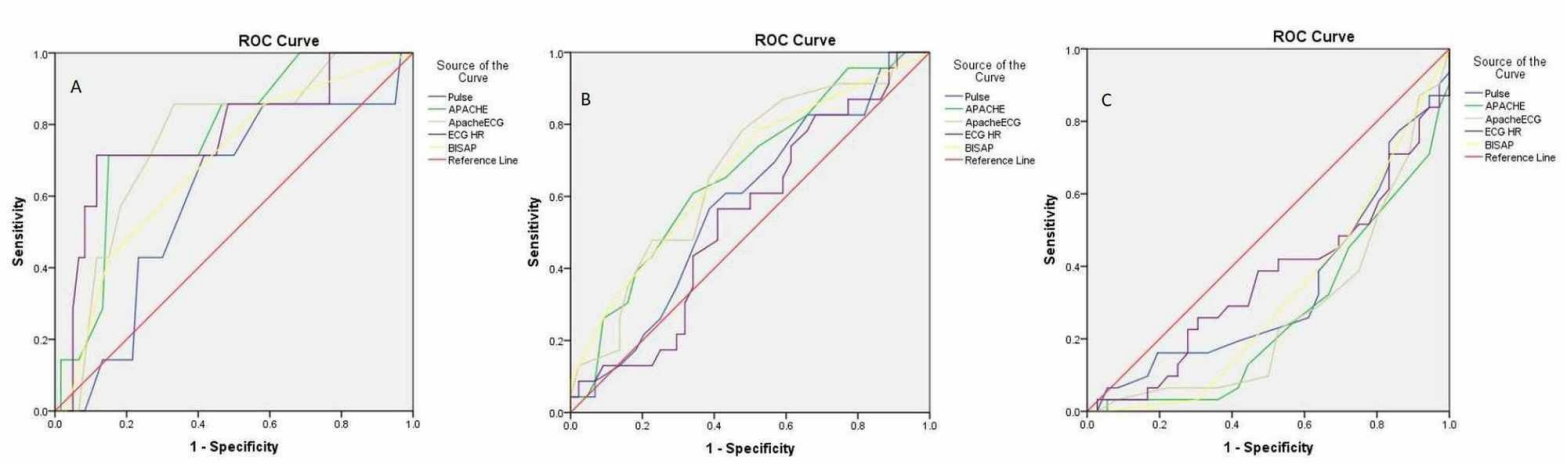
Area under the curve for severe (A), moderate (B), and mild (C) acute alcohol-related pancreatitis

ECG heart rate was the best predictor of severe pancreatitis with AUC of 0.773 (for a cut-off of 90 beats/min, with a sensitivity and specificity of 71.5% and 74.5% respectively) while E-APACHE was the best predictor for moderately severe pancreatitis (Table 4 and Figure 1).

**Table 4:** Area under the curve for pulse rate, ECG heart rate, APACHE II score, and E-APACHE score ECG, electrocardiogram; BISAP, Bedside Index for Severity in Acute Pancreatitis; APACHE, Acute Physiology and Chronic Health Evaluation

	Severe pancreatitis		Moderate pancreatitis		Mild pancreatitis	
	Area under curve	P value (CI)	Area under curve	P value (CI)	Area under curve	P value (CI)
Pulse rate	0.604	0.37(0.391-0.816	0.578	0.29(0.437-0.719)	0.340	0.02(0.207-0.473)
ECG heart rate	0.773	0.01(0.570-0.975)	0.542	0.57(0.399-0.685)	0.365	0.05(0.230-0.499)
APACHE II	0.771	0.01(0.606-0.938)	0.663	0.03(0.527-0.798)	0.263	0.001(0.144-0.381)
E-APACHE II	0.758	0.02(0.584-0.932)	0.672	0.02(0.538-0.807)	0.275	0.002(0.152-0.397)
BISAP score	0.690	0.10(0.489-0.892)	0.670	0.02(0.532-0.808)	0.318	0.01(0.191-0.444)

## Discussion

In most countries, alcohol is the second commonest cause of acute pancreatitis [9]. In developing countries, AAP causes about 30% of cases [10]. Gallstone-related pancreatitis is twice as common as AAP [11]. Studies from India report either alcohol or gall stones as the commonest reason for pancreatitis. Alcohol was the commonest cause for acute pancreatitis in studies by 50% (n=54) by Chauhan et al., 59.63% (n=123) by Negi et al., and 51% (n=110) by Vengadakrishnan et al. [12-14]. In a study from Kolkata of 234 patients, AAP constituted only 29% [15]. Their study had 63.5% with diabetes mellitus and 56.2% smokers compared to 22.5% and 29.5% in our study respectively. APACHE and BISAP scores were not calculated in any of these studies, nor was AWS a complication reported in either study. In a study by Venkatesh et al., a comparison of scoring systems in 164 patients (115 with AAP) was studied, but without the component of alcohol withdrawal [16]. Only 23% (40/168) of patients in a study from Delhi was due to AAP [17].

Friedreich first proposed the relation between alcohol and pancreatitis in 1878 [18]. Only a minority of patients drinking alcohol will develop pancreatitis, indicating a second susceptibility factor. The drinking pattern and diet are not associated with the development of pancreatitis. Obesity and inherited factors such as SPINK1 mutations have a proven association. Smoking and type of beverage have been shown/not shown to have an association in different studies [19]. Forty-one patients in our study were smokers;17/25 patients with acute on chronic pancreatitis were smokers. We did not have details regarding the diet and BMI of our patients.

About 50% of patients develop alcohol withdrawal on stopping or reducing alcohol [20]. Sixty-eight percent of our patients had withdrawal at or following admission into hospital. Coexisting gastrointestinal or respiratory disease are risk factors for withdrawal delirium. Alcohol withdrawal syndrome is characterized by tachycardia, hypertension, altered consciousness (delirium), dyselectrolytemia (potassium and sodium), and hemoconcentration (vomiting), all of which can affect severity scoring systems such as APACHE II and BISAP [20]. APACHE II and E-APACHE II scores were significantly lower in patients with AWS compared to those without (Table 1). We believe that could partly be due to younger patients, and hemodilution and anemia (CKD, cirrhosis, alcoholic hepatitis, upper gastrointestinal bleed, acute on chronic pancreatitis, and alcohol). None of the four deaths had AWS. Only one study was available that explored the relationship between AAP and alcohol withdrawal [21]. Nordback et al. hypothesized that pancreatitis was one of the manifestations occurring due to withdrawal; they found that 71% (n=100) developed pancreatitis during the first two days of withdrawal. We did not have data regarding the time of alcohol withdrawal in relation to pancreatitis [21].

The 2012 Revised Atlanta Classification is the most used system to grade pancreatitis [1]. Based on the CTSI, the frequencies among the 138 patients were 58 (0-1), 37 (2-3), 16 (4-6), and 1 (8-10). Three patients with CTSI of 4-6 and one patient with CTSI of 8 died in our study. Necrosis and pseudocyst were seen in three patients each, while ascites and pleural effusion were seen in 11 and 9 patients respectively. BISAP and APACHE II scores are commonly used to predict the severity of acute pancreatitis, the former due to its accuracy and simplicity, and the latter due to it being highly validated [1]. The accuracy of various scoring systems is comparable [9]. Eighty to 85% of patients with acute pancreatitis have mild severity that corresponded with 76% in our study [1]. In our study, APACHE II and E-APACHE-II scores did well for all the grades of pancreatitis, while BISAP had high AUC only for moderate and mild pancreatitis (Table 4).

Hypothesized reasons for myocardial involvement in pancreatitis include dyselectrolytemia, toxic effects of pancreatic enzymes, and a cardiobiliary reflex among others [22]. Cardiovascular changes in acute pancreatitis include tachycardia, hypovolemia, cardiac regional wall motion abnormalities, pericardial effusion, ventricular and atrial fibrillation, and QTc prolongation, shortened PR interval, bradycardia, ST-segment depression and elevation, and T-wave changes [18]. Only PR-interval was significant between the three groups of pancreatitis, with mild pancreatitis having significantly shorter PR-intervals (135.6±20.4 vs 142.8±21.8) compared to the others. Fifty percent of patients with pancreatitis can have ECG changes. Nadkarni et al. prospectively studied 50 patients without prior cardiac illness and found that prolonged QTc and diastolic dysfunction were predictors of mortality in acute pancreatitis. All patients who died had prolonged QTc [23]. In our study, sinus tachycardia, sinus bradycardia, prolonged QTc, and short PR-interval were seen in 31, 11, 25, and 26 individuals respectively. Among the four deaths in our study, one had sinus tachycardia and prolonged QTc each, and none had a short PR interval or sinus bradycardia. None of the four had dyselectrolytemia that could contribute to a prolonged QTc. T-wave changes were seen in three (including one Wellen’s pattern), and ST-segment changes were observed in two patients. Echocardiography could not be done in all patients, but in the seven that were performed none had pericardial effusion or diastolic dysfunction.

In a study from Turkey, ECG was studied in 64 patients with acute pancreatitis and 65% of patients had at least one electrolyte abnormality, the most common being hypokalemia [24]. In another study of 54 patients with severe acute pancreatitis and ECG findings, hypomagnesemia (n=15) was an important negative correlate of sinus tachycardia (n=15). A third of their patients had elevated CK-MB [25]. The ECG was not directly correlated with the severity scoring system. Mehmet and colleagues studied QT intervals and QT dispersion in 134 patients with acute pancreatitis, 32 of them related to alcohol. Eighty-eight of their patients had changes in ECG, with early repolarization in lateral leads being the most common finding. QT intervals were not prolonged in their patients [26]. QT interval dispersion (the difference between the maximum and minimum QT intervals) was significant between the ECGs performed during an acute attack of pancreatitis and at the time of remission [26]. In another study of 120 patients from Turkey who presented with an acute abdomen, the performance of an ECG resulted in a diagnosis needing management by the Cardiology services in 10 patients. Overall, 38 patients had ECG changes and one died [27].

Heart rate (variability) has been studied only once in severe acute pancreatitis in 41 patients (seven with AAP) with pancreatic necrosis and multiorgan dysfunction syndrome [4]. Features suggestive of sympathetic suppression during the acute phase predicted these two complications with a high AUC. Our study is the first to study heart rate as a predictor of the severity of AAP in the presence of AWS. ECG heart rate could not predict complications such as ascites, necrosis or pseudocyst formation in our study. 

Limitations

Our study had some limitations. Triglycerides and calcium were not available for all patients and hence hypertriglyceridemia as an etiological factor could not be excluded. Facility for lactate dehydrogenase was unavailable and hence Ranson scoring could not be performed. Arterial blood gases and CT abdominal findings were available only in 69/138 and 113/138 patients respectively. Detailed drug history was available for only one patient with hydrochlorthiazide use as a contributing factor for pancreatitis. Being a teaching hospital, there was an element of referral bias contributing to the number of patients with pancreatitis. This retrospective data was from a single treating Unit in the Department of Medicine. We did not access data of the other four medical units and the Surgery department which may have shown a different picture regarding the severity and mortality of AAP.

## Conclusions

Most patients with AAP appear to have mild severity of pancreatitis and more than two-thirds are associated with an alcohol withdrawal syndrome. ECG findings in AAP are also seen in half the patients, comparable to other studies of acute pancreatitis. ECG heart rate has not been previously studied in patients with both AAP and AWS and is an easy and inexpensive test to predict the severity of pancreatitis in this cohort.
